# Evaluation of Reporting Quality of Glaucoma Randomized Controlled Trial Abstracts: Current Status and Future Perspectives

**DOI:** 10.3390/life14010117

**Published:** 2024-01-12

**Authors:** Ana Vucinovic, Josipa Bukic, Doris Rusic, Dario Leskur, Ana Seselja Perisin, Marijana Radic, Marko Grahovac, Darko Modun

**Affiliations:** 1 Department of Ophthalmology, University Hospital Centre Split, Spinciceva 1, 21000 Split, Croatia; avucinovic@kbsplit.hr; 2 Department of Pharmacy, University of Split School of Medicine, Soltanska 2, 21000 Split, Croatia; drusic@mefst.hr (D.R.); dleskur@mefst.hr (D.L.); aperisin@mefst.hr (A.S.P.); marko.grahovac@mefst.hr (M.G.); dmodun@mefst.hr (D.M.); 3 Department of Neurology, General Hospital Pula, Santoriova 24a, 52100 Pula, Croatia; marijana.radic@obpula.hr

**Keywords:** glaucoma, RCT, research, CONSORT

## Abstract

The aim of this study was to explore adherence to the Consolidated Standards of Reporting Trials (CONSORT) reporting standards in abstracts of randomized controlled trials on glaucoma. A cross-sectional observational study was conducted on the aforementioned abstracts, indexed in MEDLINE/PubMed between the years 2017 and 2021. In total, 302 abstracts met the inclusion criteria and were further analyzed. The median score of CONSORT-A items was 8 (interquartile range, 7–10) out of 17 (47.0%). Most analyzed studies were conducted in a single center (80.5%) and the abstracts were predominantly structured (95.0%). Only 20.5% of the abstracts adequately described the trial design, while randomization and funding were described by 6.0% of the abstracts. Higher overall scores were associated with structured abstracts, a multicenter setting, statistically significant results, funding by industry, a higher number of participants, and having been published in journals with impact factors above four (*p* < 0.001, respectively). The results of this study indicate a suboptimal adherence to CONSORT-A reporting standards, especially in particular items such as randomization and funding. Since these factors could contribute to the overall quality of the trials and further translation of trial results into clinical practice, an improvement in glaucoma research reporting transparency is needed.

## 1. Introduction

Glaucoma is a complex multifactorial eye disease that manifests in chronic progressive optic neuropathy. This chronic progressive optic neuropathy could lead to irreversible sight loss, the most devastating consequence of this disease that has tremendous impact on affected patients’ quality of life [1]. Studies have shown that in the year 2020, more than 80 million people worldwide were affected by glaucoma, while approximately 10% of all glaucoma cases resulted in bilateral blindness. Interestingly, a study by Shih et al. suggested higher eye-related costs for open-angle glaucoma patients compared with those for ocular hypertension patients. However, the load of glaucoma on healthcare systems could be further evaluated in future studies [2,3]. Most patients suffer from open-angle glaucoma, which has a prevalence of 2.51% from age 40 to 80, and this number is expected to increase to over 112 million by 2040, imposing a major burden on global healthcare [3].

Although glaucoma is not always caused by increased intraocular pressure observed in the patients, according to the literature, increased intraocular pressure is a well-recognized modifiable risk factor of glaucoma [4]. In order to ensure the appropriate treatment of patients with increased intraocular pressure, a correct intraocular pressure measurement is crucial. Currently, it is well-known that there are several modalities to measure intraocular pressure, but Goldmann Applanation Tonometry (which is based on the Imbert–Fick law) is considered to be the gold standard in the assessment of intraocular pressure [5,6]. It should also be noted that intraocular pressure measurement could be influenced by external factors. For instance, the results of a meta-analysis by Albis-Donado et al. suggested there is a higher risk that intraocular pressure measurement could be underestimated when Goldmann Applanation Tonometry is used at higher altitudes. This is particularly the case in patients with glaucoma or those with thinner corneas and a history of corneal refractive surgery. In addition, all IOP measurement modalities, from Goldmann Applanation Tonometry to rebound tonometry, are influenced by other external factors, such as body position, central corneal thickness, corneal astigmatism, altitude, etc. [7,8].

Other important risk factors for glaucoma development described in the scientific literature include older age, not being in the White race group, and a history of glaucoma in one’s family [9]. It has also been observed that systemic disease, such as Cushing’s disease, and medication use may too predispose individuals to glaucoma. A review by Stein et al. highlighted medicines such as corticosteroids, several medications for depression, medication for epilepsy (topiramat in particular), and anticholinergics as the ones associated with glaucoma occurrence. It should be noted that vision loss caused by glaucoma could be minimized with well-timed recognition of individuals’ systemic conditions or medications that increase their risk of glaucoma. It is also very important to refer a high-risk individual for a thorough ophthalmologic examination [10,11,12]. Lately, a new modifiable glaucoma risk factor has been recognized in the scientific literature. The results of a recently published study by Sun et al., a prospective cohort study which included 409,053 participants from the United Kingdom, revealed that sleep patterns are also associated with glaucoma risk, and individuals who experience insomnia, daytime sleepiness, and snoring have a higher risk of glaucoma development, in comparison with individuals who have healthy sleeping patterns [13].

Glaucoma is often asymptomatic due to the binocular compensation of visual field defects. As a result, patients usually experience the first symptoms only in the advanced stages of the disease when there is significant damage to the visual field [2]. Therefore, making a correct and timely diagnosis and the right choice of therapy for particular patients is essential for sight preservation and improving the quality of life in affected individuals [14]. Diagnostic testing and monitoring for disease progression includes the measurement of intraocular pressure, perimetry, pachymetry, and optical coherence tomography [15]. In addition to the fundoscopic signs of glaucoma—an enlarged optic cup, an increased cup–disc ratio, the loss of the neuroretinal rim, the presence of disc hemorrhages, and parapapillary tissue atrophy—assessing the disease progression can be achieved through a spectral-domain optical coherence tomography evaluation of the optic disc and visual field analysis [16]. The primary objective of the treatment of glaucoma is to reduce the intraocular pressure in order to halt the progression of the disease [17]. The treatment of glaucoma is primarily oriented toward lowering the intraocular pressure with medicaments, lasers, or incisional glaucoma surgery [18]. Unfortunately, none of the currently available treatments can reverse glaucomatous damage to the optic nerve; however, early diagnosis and appropriate treatment can slow down the progression of the disease [19].

Since the global impact of glaucoma is so vast, it is understandable that glaucoma is among the top five most frequently studied eye diseases [20]. The amount of research on glaucoma is growing with new clinical trials bringing potential new advances in this field of medicine [21]. However, clinicians often lack time to thoroughly evaluate each new study on glaucoma as the number of studies is abundant. Therefore, it seems reasonable that abstracts, the only part of published articles freely available to clinicians worldwide, should encompass all crucial data. This inclusivity would aid clinicians in assessing the suitability and usefulness of the found abstract, thereby enabling them to filter and select articles for which they will then seek the full text to read. In order to improve research transparency and to ensure translation of research into clinical practice, reporting guidelines have been introduced. One of them, the CONSORT statement for reporting on randomized trials, was first published in 2010 in order to consolidate the standards of reporting randomized controlled trials [22]. The aforementioned statement is an evidence-based minimum set of recommendations (checklist) for reporting data from randomized controlled trials. Since the introduction of this checklist, many scientific journals have recommended its use for drafting the manuscript in order to ensure the transparent reporting of trials and their easier translation into clinical practice [23,24].

Previous studies evaluated the quality of abstracts (adherence to the CONSORT-A checklist) in several fields of medicine, but to our knowledge, the evaluation of reporting the quality of glaucoma randomized controlled trial abstracts has not yet been conducted. Since it is estimated that the number of glaucoma patients will rise and represent a public health problem, the aim of our study was to assess the adherence of glaucoma randomized controlled trial abstracts to a CONSORT-A checklist.

## 2. Materials and Methods

We conducted a cross-sectional observational study on abstracts of randomized controlled trials in the field of glaucoma, indexed in MEDLINE/PubMed. Inclusion criteria were the following: randomized controlled study design, studies with a control group, and studies with a comparison of particular intervention with either placebo intervention, active treatment, or no treatment at all. Exclusion criteria were being a cross-sectional, observational, or any other type of study except for randomized controlled trials. Animal studies and studies that did not include glaucoma patients were also excluded from our study.

The study period was from the year 2017 to 2021. The year 2017 was chosen because the guidelines used for our studies were published in 2008, and we assumed that a 10-year period would allow both authors and editors to be familiar with Consolidated Standards of Reporting Trials (CONSORT) guidelines for abstracts [11]. The year 2022 was not included as there was a possibility that some glaucoma articles would still be published during late 2022, and the exclusion of these articles could have an impact on the comparison between each study year.

**Figure 1:**
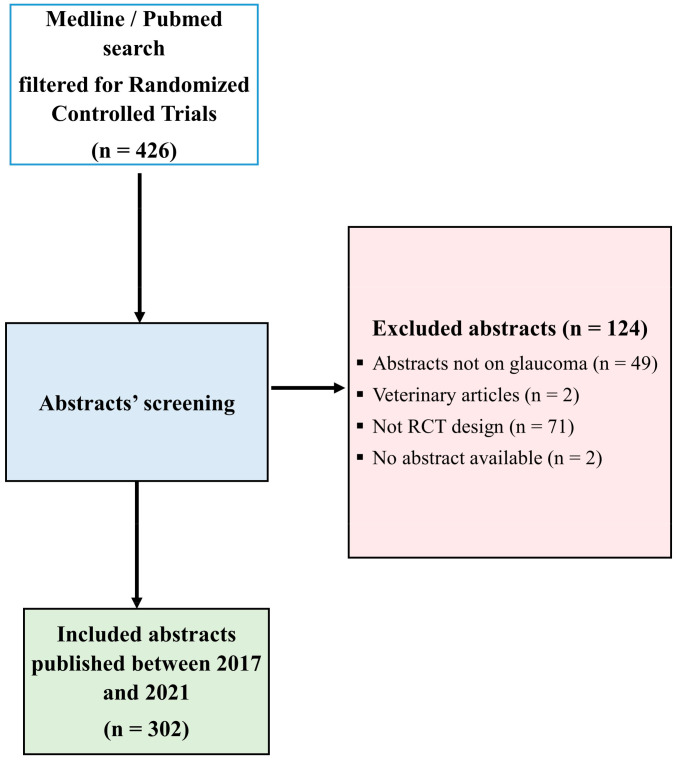
Flow diagram with search strategy and study selection. CONSORT, Consolidated Standards of Reporting Trials.

The search strategy used for article extraction in MEDLINE/PubMed consisted of searching for “*Intraocular Pressure*” [MeSH Terms] OR “*Glaucoma*” [MeSH Terms]) AND ((randomizedcontrolledtrial[Filter]) AND (2017:2021[pdat]). This data extraction method included 426 abstracts. After excluding abstracts that were not conducted on glaucoma patients or human participants, studies that were not designed as randomized controlled trials, and articles for which abstracts were not available, we analyzed 302 abstracts, all abstracts of full-text articles. Diagram flow is presented in Figure 1.

Two authors independently assessed each abstract for the inclusion of CONSORT items. One of the authors is an ophthalmology specialist with previous experience in the conduction of randomized controlled trials, and the second author is an experienced research professional with previously published articles on CONSORT matters. Disagreements between these two authors were resolved through discussion with the third author, the most experienced team member with a background in both randomized controlled trial conduction and CONSORT research.

Data were presented, where applicable, as an overall number and proportion (percent), median and interquartile range (IQR), mean and the standard deviation (SD), or mean and a 95% confidence interval (CI). The Cohen κ coefficient was used to determine interobserver agreement between the authors for individual CONSORT for abstract items. Agreement was considered sufficient for kappa point estimates higher than 0.6. To determine which factors were associated with a higher quality of reporting, a univariate linear regression analysis was conducted. The overall quality score, presented as a percentage of the total score, was a dependent variable. Those factors from the univariate analysis that were significantly associated with a higher quality (*p* < 0.05) were included in the multivariate regression analysis. The statistical analysis was conducted using SPSS (version 16.0, IBM Corporation, Chicago, IL, USA).

## 3. Results

Approximately half of the glaucoma randomized controlled trial abstracts included in this study reported the results of the pharmacological trials (132/302, 43.7%). A similar distribution was noted for the study setting, with 143/302 (47.4%) randomized controlled trials being conducted in a hospital setting, and the number of the participants, with 132/302 (43.7%) randomized controlled trials including more than 100 participants. Most of the studies described in the abstracts were conducted in a single center (243/302, 80.5%), and they had reported results where the main outcome measure was significantly different from the control (194/302, 64.2%). Abstracts were predominantly structured (287/302, 95.0%). On average, abstracts had a median of six authors (IQR 4–8). The mean impact factor of the journals in which they were published was 5.73 (SD = 17.42). A full description of the study characteristics is provided in Table 1.

The interobserver agreement in the present study was found to be sufficient as the calculated Cohen κ values for all items were above the recommended threshold value of 0.6, as presented in Table 2.

Only 62 (20.5%) of all the included abstracts adequately described the trial design (e.g., parallel, crossover, etc.). A proper title and the corresponding author details were provided by 118 and 136 (39.1% and 45.0%) of the included abstracts, respectively. With regards to the methodology section, interventions and objective items were overwhelmingly well-reported (294/302, 97.4% and 296/302, 98.0%, respectively). On the contrary, randomization and blinding were poorly reported by most abstracts, with only 18 (6.0%) and 56 (18.5%) sufficiently describing those items. Participant items were properly described in 186 (61.6%) abstracts, while the outcome was reported in 222 (73.5%) abstracts.

Results of the primary outcome measures were adequately described in 236 (78.1%) abstracts. The larger proportion of the included abstracts (224, 74.2%) provided the number of participants randomized in each trial group; however, most abstracts failed to report the number of participants included in the analysis, with only 61 (20.2%) abstracts providing this valuable information. Another poorly reported item was the side effects and adverse events, which were described in only 87 (28.8%) of the included abstracts. Funding statement was provided by only 18 (6.0%) abstracts. Fifty abstracts (16.6%) gave the trial registration information. Adequate conclusions were provided by the vast majority of the abstracts (295, 97.7%). The adherence of each item to the CONSORT for the abstract guideline is provided in Table 3.

Only one abstract adequately reported every item and had a maximum score of 17. The median score was 8 (IQR 7–10) out of 17 (47.0%) items. The average overall reporting scores of the included abstracts are described in Table 4.

The overall quality score for each study characteristic of interest that was included in the regression analysis is presented in Table 5.

Table 6 presents the results of the linear regression analysis. Cutoff values of 2.200 and 4.000 for journal impact factors were chosen to create three approximately equal groups. This decision was made to facilitate a balanced categorization of journals based on their impact factors. The goal was to have comparable representation in each group, which enhanced the robustness of the analysis and provided a more nuanced understanding of the data.

In a univariate model, the higher overall scores were associated with structured abstracts (*p* < 0.001), pharmacological intervention (*p* < 0.01), a multicenter setting (*p* < 0.001), statistically significant results (*p* < 0.001), funding by the industry (*p* < 0.001), a higher number of participants (*p* < 0.001), having been published in journals with impact factors above 4 (*p* < 0.001) and in journals in the first three quartiles, as well as having more authors, namely above seven (*p* < 0.01), and being authored by a collaboration (*p* < 0.001). As no significant association was found with the study setting, it was omitted from the multivariate analysis. A higher overall score remained associated with structured abstracts (*p* < 0.001), a multicenter setting (*p* < 0.05), statistically significant results (*p* < 0.05), industry funding (*p* < 0.01), being published in the second (*p* < 0.01) and third quartile (*p* < 0.05), and having been published in journals with impact factors above four (*p* < 0.01) in the multiple regression model. The overall score was adversely impacted by being published in journals with impact factors between 2.201 and 4.

## 4. Discussion

This study analyzed 302 abstracts of glaucoma randomized controlled trials and found scarce reporting of CONSORT-A checklist items, as the majority of abstracts included mostly half of all items. It should be noted that only one abstract included all the checklist items. Further, items such as randomization and funding were reported in less than 10% of all abstracts. The results of our study should raise awareness of the current status of the reporting quality in the field of glaucoma. Since this is a rising field, inadequate reporting quality could have an impact on the design of future studies and also on the translation of current conclusions into clinical practice.

The results of our study showed that adverse effects were reported in merely 30% of glaucoma randomized controlled trial abstracts. Since it could be assumed that clinicians did not have time to read through the whole manuscript in search of high-quality information for their patients, the omission of adverse effect data in abstracts could lead to inappropriate intervention in particular patients. In general, due to both time limitations and very often, the non-availability of the full-text articles, most clinicians and scientists perform the preliminary evaluation of the quality and validity of a clinical trial just by screening the abstracts. Therefore, it is of great importance that the abstract is well-written, detailed, and transparent, as this eliminates bias and confounding factors.

The results of our study were in accordance with the results of several previous studies, which reported a low adherence to the CONSORT-A guidelines [25,26,27]. Song et al. reported that although the reporting quality of psychiatry randomized controlled trial abstracts improved after the publication of the CONSORT-A guidelines, it still remained suboptimal with an overall quality score of just 45% [25]. Janackovic et al. reported that randomized controlled trials published in the seven top anesthesiology journals did not adhere to CONSORT-A guidelines with a median adherence of only 41% [26]. Baulig et al. reported that none of the 136 investigated abstracts on macular degeneration reported all of the CONSORT-A items, and the median number of reported items was seven [27].

A number of authors showed that there was substandard reporting of funding in randomized controlled trial abstracts, also observed in our study, which could be misleading to the reader, as it is well-known that funding by industry could be associated with the positive results of randomized controlled trials [28,29]. Previous research has also demonstrated that inaccurate or omitted funding information could lead to the uncritical incorporation of those results into clinical practice [30]. Fundytus et al. showed that the vast majority of oncology randomized controlled trials are now funded by industry—they are larger, more likely to be positive, and published in higher impact journals than trials without industry support [31]. Furthermore, the results of a study by Wiehn et al. showed that adequate reporting varied considerably across CONSORT items with information on blinding and adverse effects being the least reported [32]. Although the results of several studies have concluded that funding is mostly not included in the abstract of randomized controlled trials, it has been observed that funding is reported in the full text of the randomized controlled trial articles. For instance, a study by Alharbi et al. reported that funding was included in 76.9% of periodontal studies, published in three of the most citable journals in the field. This proportion was more than ten times higher when compared to our results. However, there is a possibility that the majority of journals include additional sections for acknowledgements, conflicts of interest, and funding. Nonetheless, as mentioned before, not all scientific articles are freely available, and readers should be able to distinguish between funded and non-funded research based on the abstract data [33].

Improving the reporting quality of randomized controlled trial abstracts requires concerted efforts and considerations. Firstly, addressing the limitation imposed by word count restrictions on abstract length is crucial. Journals should reconsider these limitations, recognizing that comprehensive reporting may necessitate additional space. Secondly, promoting structured abstracts is paramount, given our findings on their significantly better reporting quality. Journals not endorsing structured abstracts should consider adopting this practice, while those already doing so could refine the designated structure to align more closely with CONSORT-A guidelines. Thirdly, educating authors on the effective utilization of CONSORT-A guidelines for abstract writing is imperative. Workshops and resources should be provided to enhance authors’ understanding and application of these guidelines. Additionally, educating peer reviewers to encourage authors to structure their abstracts in accordance with CONSORT-A guidelines is essential for maintaining reporting standards. Furthermore, editors should play a proactive role by insisting on stricter adherence to these guidelines during the review process. Finally, journals should explicitly endorse CONSORT-A guidelines in their submission guidelines for authors, reinforcing the importance of compliance. Stricter enforcement mechanisms should be implemented to ensure that these guidelines become an integral part of the abstract writing process, fostering a culture of comprehensive reporting in the field of glaucoma research.

It is worth mentioning that published glaucoma articles, or their available abstracts, could be used as study materials for both students during their formal education and the general population while initiating public health interventions. For instance, an advanced approach to formal education for medical students was described by Marin et al. The authors provided valuable data on experience with a pilot model of ophthalmology longitudinal integrated clerkships, which improved students’ knowledge in this field, but the authors also recognized the need for future studies that would evaluate the relationship between medical curricula and students’ interest in ophthalmology. Similarly, a need for accurate information that would allow glaucoma patients to make informed decisions on their condition was recognized in a study by Cohen et al., where authors evaluated patient education materials available online [34,35]. Therefore, in order to ensure that study materials are of high quality, items such as funding, but also harms and outcomes, must be represented in article abstracts.

The limitation of this study was that we used only one database—MEDLINE/PubMed—because of the fact that it is the most used database for medical professionals worldwide and it is a free-access database. The strengths of this study were its reproducibility and selection criterion transparency, as well as a wide timeframe from 2017 to 2022. Finally, we would like to emphasize that the interobserver agreement measured by Cohen’s kappa was adequate in all the checklist items.

## 5. Conclusions

Our study indicated that the publication of the CONSORT-A guidelines has not yet translated into better randomized controlled trial abstract reporting in the field of glaucoma research. Glaucoma is a common and serious condition with an increasing need for high-quality, evidence-based information; however, the reporting quality of randomized controlled trial abstracts concerning glaucoma had not been assessed until this study was conducted. Since items included in the abstracts could assist the evaluation of the quality of the presented trials and the further translation of the trial results into clinical practice, an improvement in glaucoma research reporting transparency is needed. Further efforts on implementing the guidelines are required to enhance the quality of reported data and facilitate the translation of scientific research into clinical practice.

## Figures and Tables

**Table 1:** Characteristics of included abstracts.

Characteristics	N	%
**Type of intervention**		
Non-pharmacological	170	56.3
Pharmacological	132	43.7
**Study centers**		
Single center	243	80.5
Multicenter	59	19.5
**Significance of results**		
Non-significant	108	35.8
Significant	194	64.2
**Number of participants**		
<100	170	56.3
≥100	132	43.7
**Funding**		
Non-industry	292	96.7
Industry	10	3.3
**Setting**		
Non-hospital	159	52.6
Hospital	143	47.4
**Abstract structure**		
Unstructured abstract	15	5.0
Structured abstract	287	95.0
**Quartiles**		
Non-ranked	38	12.6
1st	91	30.1
2nd	72	23.8
3rd	79	26.2
4th	22	7.3
	**Mean (SD)**	**Median (IQR)**
**Number of authors**	6.54 (3.41)	6.00 (4.00–8.00)
**Impact factor**	5.73 (17.42)	2.97 (2.02–5.49)

**Table 2:** Interobserver agreement for abstract reporting items.

Item	Kappa Point	Kappa > 0.60
Title	0.888	*
Authors	0.953	*
Trial design	0.791	*
**Methods**		
Participants	0.958	*
Interventions	0.607	*
Objective	0.722	*
Outcome	0.864	*
Randomization	0.607	*
Blinding	0.684	*
**Results**		
Numbers randomized	0.637	*
Recruitment	0.954	*
Numbers analyzed	0.969	*
Outcome	0.737	*
Harms	0.639	*
Conclusions	0.607	*
Trial registration	0.895	*
Funding	0.830	*

* Kappa > 0.60.

**Table 3:** Quality of the individual CONSORT for abstract items.

Items	N	%
Title	118	39.1
Authors	136	45.0
Trial design	62	20.5
**Methods**		
Participants	186	61.6
Interventions	294	97.4
Objective	296	98.0
Outcome	222	73.5
Randomization	18	6.0
Blinding	56	18.5
**Results**		
Numbers randomized	224	74.2
Recruitment	143	47.4
Numbers analyzed	61	20.2
Outcome	236	78.1
Harms	87	28.8
Conclusions	295	97.7
Trial registration	50	16.6
Funding	18	6.0

**Table 4:** Overall reporting quality score.

	Score	Score (%)
**Mean**	8.28	48.32
**SD**	2.34	13.61
**95% CI**	8.02–8.55	46.77–49.86
**Median**	8.00	47.00
**IQR**	7.00–10.00	41.00–58.00

**Table 5:** Overall reporting quality score for each study characteristic.

Characteristics	Mean Score (%)	95% CI
**Type of intervention**		
Non-pharmacological	46.45	44.44–48.45
Pharmacological	50.72	48.35–53.09
**Study centers**		
Single center	46.12	44.59–47.66
Multicenter	57.34	53.29–61.39
**Significance of results**		
Non-significant	44.60	42.00–47.20
Significant	50.38	48.51–52.25
**Number of participants**		
<100	45.12	43.29–46.95
≥100	52.42	49.95–54.90
**Funding**		
Non-industry	47.59	46.10–49.07
Industry	69.60	56.94–82.26
**Number of authors**		
<5	44.63	41.90–47.36
5–7	45.10	42.85–47.35
>7	51.40	48.21–54.59
Collaboration	57.38	52.82–61.93
**Setting**		
Non-hospital	47.79	45.56–50.01
Hospital	48.90	46.77–51.04
**Abstract structure**		
Unstructured abstract	34.53	22.54–46.53
Structured abstract	49.04	47.56–50.52
**Impact factor**		
<2.200	42.69	40.13–45.24
2.201–4	44.51	42.60–46.41
>4	56.60	53.96–59.23
**Quartiles**		
Non-ranked	39.00	35.21–42.79
1st	55.28	52.60–57.95
2nd	50.06	46.33–53.74
3rd	44.46	42.29–46.62
4th	43.77	39.76–47.79

**Table 6:** Linear-regression-derived estimates and 95% CI with the dependent variable defined as the mean overall quality score shown as a percentage.

Characteristics	Univariate Analysis, Estimate 95% CI	Multivariate Analysis, Estimate 95% CI
**Type of intervention**		
Non-pharmacological	Reference	Reference
Pharmacological	4.273 (1.198–7.347) **	2.406 (−0.170–4.982)
**Study centers**		
Single center	Reference	Reference
Multicenter	11.216 (7.536–14.895) ***	4.367 (0.822–7.911) *
**Significance of results**		
Non-significant	Reference	Reference
Significant	5.780 (2.626–8.933) ***	2.946 (0.335–5.557) *
**Number of participants**		
<100	Reference	Reference
≥100	7.301 (4.300–10.301) ***	2.424 (−0.304–5.152)
**Funding**		
Non-industry	Reference	Reference
Industry	22.014 (13.756–30.273) ***	12.341 (4.775–19.907) **
**Number of authors**		
<5	Reference	Reference
5–7	0.466 (−3.347–4.279)	0.686 (−2.597–3.968)
>7	6.770 (2.767–10.772) **	2.358 (−1.152–5.869)
Collaboration	12.742 (7.816–17.669) ***	3.339 (−1.570–8.248)
**Setting**		
Non-hospital	Reference	
Hospital	1.116 (−1.974–4.206)	
**Abstract structure**		
Unstructured abstract	Reference	Reference
Structured abstract	14.502 (7.588–21.415) ***	14.784 (8.867–20.700) ***
**Impact factor**		
<2.200	Reference	Reference
2.201–4	1.820 (−1.617–5.257)	−4.277 (−8.521- (−0.032)) *
>4	13.912 (10.535–17.288) ***	11.327 (4.396–18.259) **
**Quartiles**		
Non-ranked	Reference	Reference
1st	16.275 (11.533–21.016) ***	−0.701 (−8.894–7.491)
2nd	11.056 (6.133–15.978) ***	8.299 (2.820–13.778) **
3rd	5.456 (0.609–10.302) *	6.943 (1.464–12.423) *
4th	4.773 (−1.804–11.350)	2.820 (−2.933–8.572)

* *p* < 0.05, ** *p* < 0.01, *** *p* < 0.001.

## Data Availability

The data are available upon reasonable request to the corresponding author.
